# Reversible thermal unfolding of a yfdX protein with chaperone-like activity

**DOI:** 10.1038/srep29541

**Published:** 2016-07-11

**Authors:** Paramita Saha, Camelia Manna, Jaydeb Chakrabarti, Mahua Ghosh

**Affiliations:** 1 Department of Chemical, Biological And Macromolecular Sciences, S. N. Bose National Centre for Basic Sciences, Block JD, Sector III, Salt lake, Kolkata 700106, India

## Abstract

yfdX proteins are ubiquitously present in a large number of virulent bacteria. A member of this family of protein in *E. coli* is known to be up-regulated by the multidrug response regulator. Their abundance in such bacteria suggests some important yet unidentified functional role of this protein. Here, we study the thermal response and stability of yfdX protein STY3178 from *Salmonella* Typhi using circular dichroism, steady state fluorescence, dynamic light scattering and nuclear magnetic resonance experiments. We observe the protein to be stable up to a temperature of 45 °C. It folds back to the native conformation from unfolded state at temperature as high as 80 °C. The kinetic measurements of unfolding and refolding show Arrhenius behavior where the refolding involves less activation energy barrier than that of unfolding. We propose a homology model to understand the stability of the protein. Our molecular dynamic simulation studies on this model structure at high temperature show that the structure of this protein is quite stable. Finally, we report a possible functional role of this protein as a chaperone, capable of preventing DTT induced aggregation of insulin. Our studies will have broader implication in understanding the role of yfdX proteins in bacterial function and virulence.

Complete genome sequencing of several organisms have resulted in new proteins^1^ many of which are neither functionally nor structurally characterized. Among such proteins, yfdX proteins are ubiquitously present in virulent gram negative bacteria^2^. Although yfdX protein is identified many years back^3^,^4^, detailed characterization of both function and structure is lacking. The only structural information (PDB 3DZA) on this protein is from *K. pneumoniae* where no function is reported. An earlier study on yfdX protein from *E. coli* reports its up-regulation and co-expression with a regulatory protein evgA, involved in multidrug resistance^3^,^4^,^5^. Our earlier study^2^ on yfdX protein SYY3178 from *Salmonella* Typhi reports its interaction with antibiotics namely ciprofloxacin, rifampin and ampicillin. This indicates that yfdX protein might be playing functional role in bacteria.

The stability of protein structure could throw light towards understanding its functionality. The structural stability gets affected by temperature shocks, pH jumps, denaturing agents such as chaotropes, alcohols or acids and results in unfolding^6^,^7^,^8^,^9^,^10^ of the protein. The thermal response for different proteins is diverse. There are many which can tolerate high temperatures, many of which unfold at elevated temperatures but refolds back to the native structure upon cooling^11^,^12^,^13^,^14^,^15^,^16^,^17^,^18^,^19^,^20^ and other form irreversible aggregates upon heating. For example, polymerases like Taq^21^ or pfu^22^ from thermophillic bacteria show very high temperature stability. Another class of protein, known as the heat shock proteins (Hsp), like IbpB^23^ from *E. coli*, plant cytosolic small Hsp of class I and II^24^, GroES^25^, to name only a few, are produced at elevated temperatures. They not only possess chaperone activity but also show thermal reversibility in unfolding. Likewise, reversibility in thermal unfolding is shown in by other chaperones, like Skp^26^ from periplasm or SlyD^27^ and SecB^28^ from cytosol of *E. coli*.

Here, we study the thermal responses of the yfdX family protein STY3178. The primary objective of this work is to shed light on its function by characterizing thermal unfolding-folding and stability. Our experimental studies unravel reversible thermal unfolding of this protein. We show the thermal stability of the protein at elevated temperatures from circular dichroism (CD), steady state fluorescence and nuclear magnetic resonance (NMR), whereas the state of oligomerization remains invariant as observed from DLS. We also measure the kinetics of thermal unfolding and refolding from CD. A model of STY3178 based on the homology and molecular dynamics (MD) simulation is proposed to understand the observed thermal stability. Finally, we identify the capability of STY3178 to act as chaperone *in vitro*.

## Results

**Figure 1:**
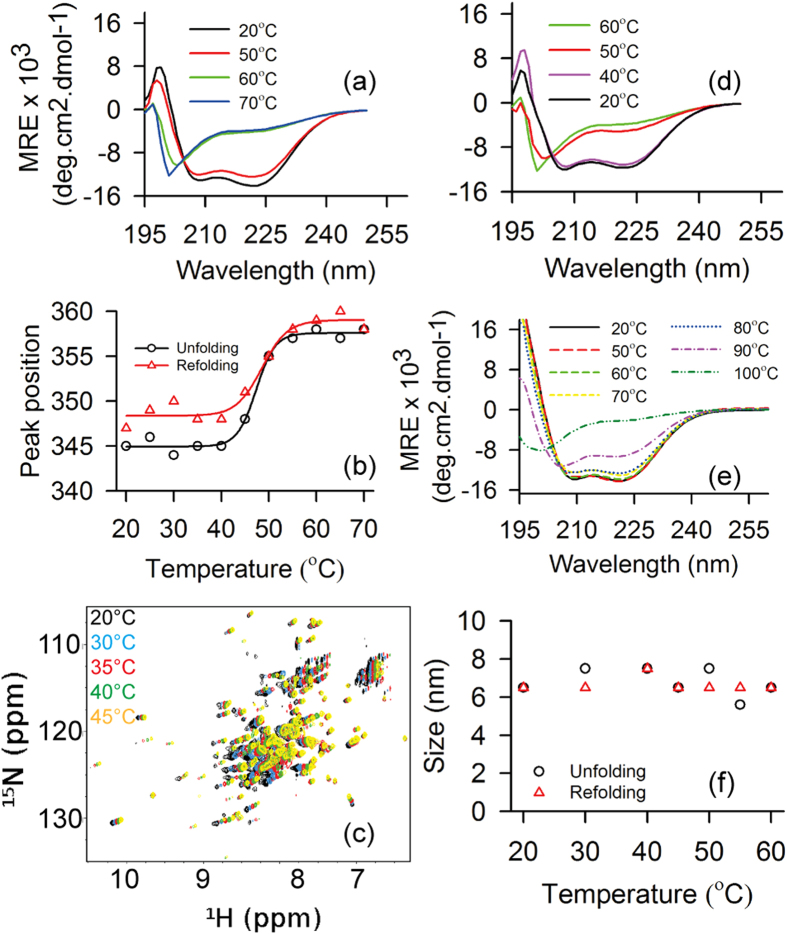
Structural changes of STY3178 at higher temperature. (**a**) Shows the CD spectra of protein for temperatures 20 °C (black), 50 °C (red), 60 °C (green) and 70 °C (blue) during heating. (**b**) Shows the change in fluorescence emission maxima for 280 nm excitation with temperature during unfolding (black) and refolding (red). (**c**) Shows the ^15^N-^1^H HSQC spectra (6–10.5 ppm) of the protein for temperatures 25 °C (black), 30 °C (blue), 35 °C (red), 40 °C (green) and 45 °C (yellow). (**d**) Shows the CD spectra upon cooling (refolding) at temperatures 60 °C (green), 50 °C (red), 40 °C (magenta) and 20 °C (black). (**e**) Far UV-CD spectra of the refolded protein recorded at room temperature after heating it for 30 minutes at 50 °C (red), 60 °C (green), 70 °C (yellow), 80 °C (blue), 90 °C (magenta) and 100 °C (dark green). (**f**) Hydrodynamic diameters of STY3178 measured from DLS plotted for temperatures 20 °C, 30 °C, 40 °C, 50 °C and 60 °C during heating (black) and cooling (red).

Circular dichroism (CD) spectrum indicates that STY3178 is an α-helical protein containing two minima around 209 and 222 nm (Fig. 1a, black). The steady state fluorescence emission maximum of this protein is around 342 nm for excitation wavelengths of 275, 280 and 295 nm. This is in agreement to our earlier observation^2^. We probe the thermal unfolding, refolding upon cooling and conformational stability of STY3178 using CD, fluorescence, DLS and NMR.

### Temperature induced unfolding and conformational stability

The α-helical secondary structure remains stable and nearly unchanged up to 50 °C when heated gradually in steps of 10 °C starting from 20 °C (Fig. 1a). The structural signature significantly changes beyond 50 °C. The two minima at 209 and 222 nm disappear completely at 60 °C (Fig. 1a) indicating total loss of secondary structure. No further change in the CD spectrum is observed at 70 °C (Fig. 1a) when compared to 60 °C spectrum (Fig. 1a). Thus we find the protein to unfold completely at 60 °C.

Next we monitor the emission maxima position for excitation at 280 nm in steady state fluorescence. The emission peak position of the protein remains unaltered till 45 °C (Fig. 1b), beyond which the maxima starts shifting towards higher wavelength. A jump of nearly 10 nm in the emission peak position is observed between 45 °C to 55 °C. This shift towards higher wavelength is an indication of protein unfolding. Beyond 55 °C, the emission peak position does not change any further. We observe a decrease in fluorescence emission intensity with increase in temperature. These observations are qualitatively similar to those from CD measurement.

HSQC spectrum of ^15^N-labelled protein shows well dispersed peaks between 6–10.5 ppm at 25 °C (Fig. 1c). We observe large number of peaks clustered around 7.5–8.5 ppm region as found for α-helical proteins in the HSQC spectrum (Fig. 1c). The spectrum also contains well dispersed peaks other than the clusters observed. This indicates presence of some β-sheet elements in STY3178. The dispersion of the HSQC spectrum changes marginally when we increase temperature from 25 °C to 45 °C. This suggests that the tertiary structural fold is indeed stable at 45 °C, in agreement with CD and fluorescence data. There are only a few peaks in the HSQC spectra which show sensitivity upon increasing temperature (Fig. 1c). Interestingly most of the peaks showing sensitivity to higher temperature belong to the well dispersed regions and not the core helical region.

### Reversibility in unfolding

We check the reversibility in unfolding by monitoring the refolding using CD by cooling down the protein from an elevated temperature. The thermally unfolded protein at 70 °C is gradually cooled to 20 °C by decreasing temperature in step of 10 °C. We observe the protein remains unfolded up to 50 °C (Fig. 1d). We observe refolding of the protein where the α-helical secondary structure is regained upon further cooling down to 40 °C (Fig. 1d). The CD spectrum after cooling down to 20 °C (Fig. 1d) is very similar to the native protein spectrum (Fig. 1a) prior to thermal unfolding. This observation demonstrates reversibility in thermal unfolding of STY3178. We observe a blue shift ~8 nm compared to the unfolded state in the fluorescence emission peak position upon cooling (Fig. 1b). This again indicates protein refolding where a native-like structure is formed which results in blue shift compared to the unfolded protein. We also observe an increase in fluorescence intensity upon refolding in contrast to the decrease during unfolding. However, the final emission peak intensity is less and the position is red shifted by 2–3 nm in the refolded protein compared to the native, suggesting slight rearrangement in side chains conformations. Overall both steady state fluorescence and CD results are in agreement indicating reversibility in thermal unfolding of this protein.

The conformational stability under equilibrium condition is probed by heating the protein for a longer period of time. We heat the protein at different high temperature water baths (50 °C to 100 °C) for 30 minutes and cool down it to room temperature after that. The CD spectra of the cooled proteins are then compared with the native one. We find that protein when heated within the range 50 °C to 80 °C, refolds completely as native-like structure, shown in Fig. 1e. There is a slight decrease in ellipticity of the refolded protein when it is treated at 90 °C, indicating partial loss of structure (Fig. 1e). We further observe that no refolding could be achieved when the protein is heated at 100 °C (Fig. 1e). Thus, the reversibility of folding is maintained in STY3178 fully when treated at 80 °C.

We measure the hydrodynamic size of STY3178 in the temperature range 20 °C to 60 °C (Fig. 1f) using DLS. The folded protein in solution at 20 °C has a hydrodynamic diameter around 6.5 nm as reported earlier^2^. The variation of this size with temperature is only around 1 nm which is within the fluctuation limit. This indicates that the oligomerization state of the protein does not change upon heating and remains stable. When we cool the same protein from 60 °C to 20 °C, we again observe similar hydrodynamic sizes (Fig. 1f).

### Heating or cooling rate dependence

**Figure 2:**
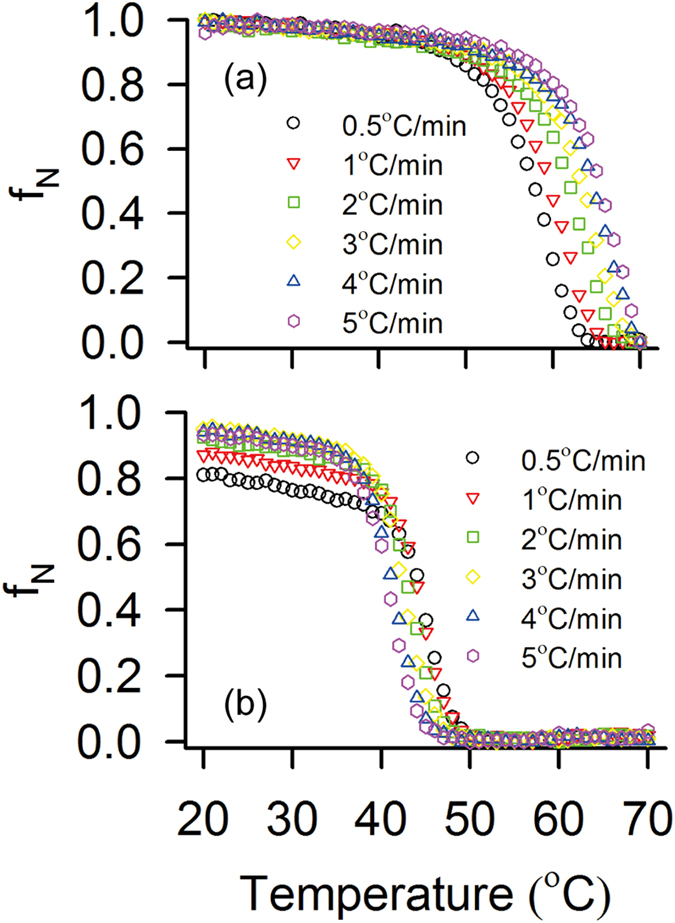
Unfolding and refolding of STY3178. (**a**) Thermal unfolding of STY3178 showing the variation of *f*_*N*_ with temperature for different heating rates 0.5 °C/min (black), 1 °C/min (red), 2 °C/min (green), 3 °C/min (yellow), 4 °C/min (blue) and 5 °C/min (magenta). (**b**) Change in *f*_*N*_ upon cooling the protein from 70 °C at different cooling rates 0.5 °C/min (black), 1 °C/min (red), 2 °C/min (green), 3 °C/min (yellow), 4 °C/min (blue) and 5 °C/min (magenta).

We perform ellipticity measurements for different heating rates. The fraction of folded protein (*f*_*N*_) is estimated from the ellipticity at 222 nm [*θ*_222_] for each temperature (as described in method section). For none of the heating rates below 50 °C, protein unfolds and *f*_*N*_ remains constant. Above 50 °C, *f*_*N*_ decreases for various rates (Fig. 2a). We observe a difference in half denaturation temperature (T_m_) that is at *f*_*N*_ = 0.5 for the different heating rates. T_m_ is low for slower heating rates and increases for faster heating rate as tabulated in Table 1. In other words, longer the protein remains at elevated temperature above 50 °C, faster is the unfolding. We also monitor the refolding of the protein using different cooling rates. The plot of *f*_*N*_ versus temperature for different cooling rates (Fig. 2b) show refolding of the protein starts when the temperature is below 50 °C. The half renaturation temperature (T_m_’) of the protein is thus lower than the half denaturation temperature (T_m_) (Table 1), indicating hysteresis in the unfolding and refolding. These observations indicate that the insufficient time during faster scan in temperature hinders the protein to come to equilibrium at a given temperature causing the hysteresis. This is also indicative of kinetically controlled process^29^,^30^,^31^. The hysteresis decreases with decreasing rates of heating and cooling.

### Kinetics of unfolding and refolding

**Figure 3:**
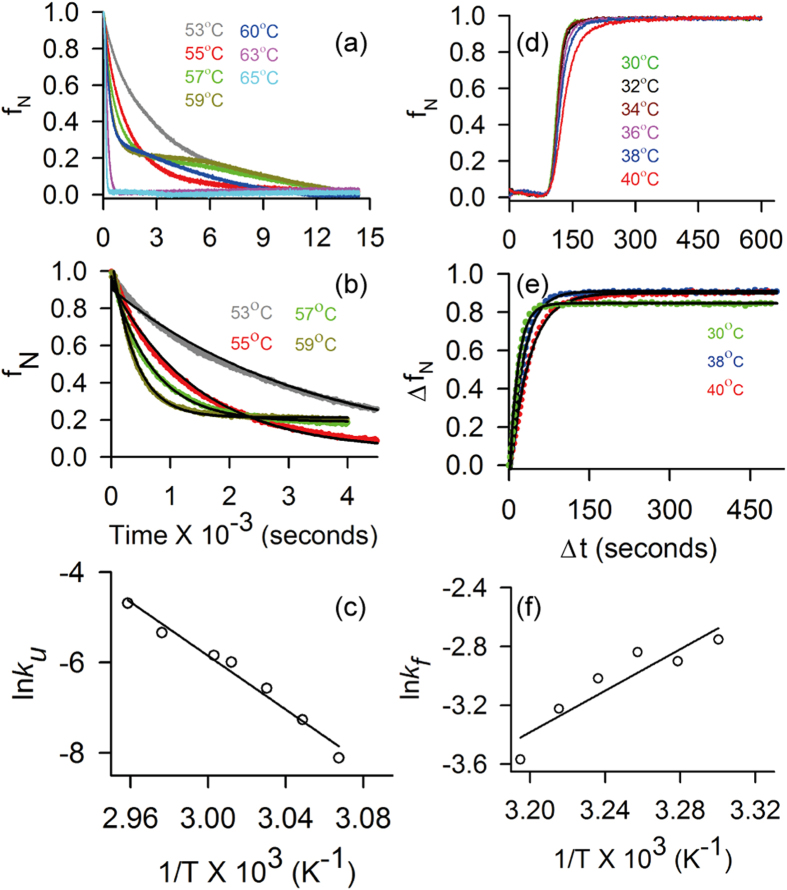
Kinetics of STY3178 unfolding and refolding. (**a**) Shows the folded protein fraction (*f*_*N*_) as a function of time upon heating for temperatures 53 °C (grey), 55 °C (red), 57 °C (green), 59 °C (dark yellow), 60 °C (blue), 63 °C (magenta) and 65 °C (cyan). (**b**) Shows exponential fitting of *f*_*N*_ with time for temperatures 53 °C (grey), 55 °C (red), 57 °C (green) and 59 °C (dark yellow). (**c**) Arrhenius plot showing the linear dependence of ln*k*_*u*_ over 1/T (Kelvin^−1^). (**d**) Shows the variation of *f*_*N*_with time during protein refolding for temperatures 30 °C (green), 32 °C (black), 34 °C (brown), 36 °C (magenta), 38 °C (blue) and 40 °C (red). (**e**) The change in *f*_*N*_ (Δ*f*_*N*_ = *f*_*N*_ − *f*_*R*_, where *f*_*R*_ is the folded fraction from which the rise in *f*_*N*_ starts) versus time (∆*t*) at which *f*_*N*_starts increasing, is fitted exponentially and shown for temperatures 30 °C (green), 38 °C (blue) and 40 °C (red). (**f**) The plot of ln*k*_*f*_ over 1/T (Kelvin^−1^) shows linear dependence.

The kinetics of unfolding of STY3178 is measured from *f*_*N*_ at a given temperature with time in the temperature range 53 °C to 65 °C (see methods) where the *f*_*N*_ values tend to zero after sufficiently long time (Fig. 3a). *f*_*N*_ decreases below 50% within approximately 50 minutes when the protein is heated in this temperature range. The plot of *f*_*N*_ with time follows single exponential decay. We obtain the rate of unfolding (*k*_*u*_) from the exponential fit of *f*_*N*_ versus time (few representative cases shown in Fig. 3b). We observe that *k*_*u*_ values follow the Arrhenius behavior as can be seen from ln*k*_*u*_ versus 1/T plot, where T is the absolute temperature (Fig. 3c). The activation energy of unfolding (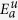) is calculated from the slope of the plot. Here 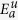 ~ 246.9 kJ/mol obtained for STY3178 is comparable to the activation of energy of unfolding reported in literature^32^,^33^,^34^. In the temperature range below 53 °C the protein does not reach complete unfolded state despite long measurement time. Hence this region has not been considered for estimating 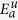.

We monitor the refolding kinetics of the protein from the change in ellipticity at 222 nm [*θ*_222_] starting from the thermally unfolded state at 70 °C. The unfolded protein is then directly cooled down to different temperatures in the range of 40 °C to 30 °C. There is a time lag (t_L_) of about 100 seconds after which *f*_*N*_ increases rapidly to reach the folded state (*f*_*N*_ ≈ 0.95) within approximately 300 seconds. The data for the entire time of measurement (0 to 600 seconds), averaged over repeated set of experiments, are shown in Fig. 3d for different temperatures. The finite t_L_ is probably due to stabilization after temperature quenching and depends slightly on temperature. We calculate Δ*f*_*N*_ = *f*_*N*_ − *f*_*R*_, where *f*_*R*_ is the folded fraction at *t* = *t*_*L*_. The *f*_*R*_ values are typically small but non zero for different temperatures. Similarly, we define the rise time Δ*t* = *t* − *t*_*L*_. The plot of Δ*f*_*N*_ versus Δt show single exponential rise, few of which are shown in Fig. 3e. We calculate the rate of refolding (*k*_*f*_), from the fitted curves. Figure 3f shows ln*k*_*f*_ versus 1/T plot confirming the Arrhenius behavior upon refolding. The activation energy (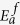) of refolding is ~−58.66 kJ/mol.

The quality of linear fit in refolding kinetics (Fig. 3f) is somewhat poorer than that in unfolding (Fig. 3c). This is reflected in the R^2^ values of the fits (0.97 for unfolding and 0.85 for refolding). This leads us to check the dependences of ln(*k*_*u*_/T) and ln(*k*_*f*_/T) on 1/T as suggested in ref. 20. The plots, shown in the Supplementary Fig. S1, confirm linear dependences for both the cases. This suggests that the activation heat capacities^20^ for both unfolding and refolding are negligible.

### Molecular modeling

**Figure 4:**
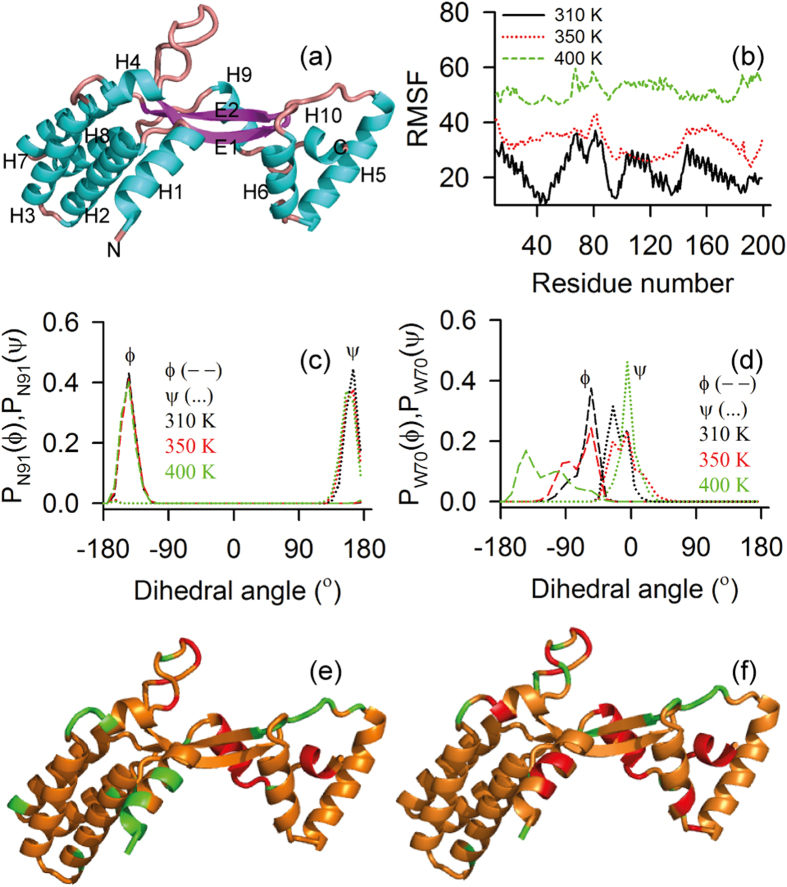
Molecular model and dihedral angle fluctuation of STY3178. (**a**) Cartoon representation of molecular model for STY3178. Ten α-helices (H1-H10, cyan) and two β-strands (E1 and E2, magenta) are marked along with the N- and C-terminals. (**b**) Root mean square fluctuation (RMSF) of each residue of the model in the equilibrated ensemble between 150–300 ns of simulated structure for temperatures 310 K (black), 350 K (red) and 400 K (green) temperatures. (**c,d**) Show the distribution of dihedral angles ϕ (dash) and ψ (dotted) at 310 K (black), 350 K (red) and 400 K (green) for residues N91and W70 respectively. (**e,f**) Show cartoon representation of the molecular model with residues as per the different degree of fluctuation at 350 K (r_350_) and 400 K (r_400_) respectively. Green indicates residues with r_350_, r_400_ ≤ 1, orange shows residues with r_350_ and r_400_ in the range 1–4 and the residues with r_350_, r_400_ > 4 are shown in red.

In the absence of a molecular structure, we propose a homology model of STY3178 to understand the stability of the protein, based on the template yfdX protein structure (PDB 3DZA) from *K. pneumoniae*. The sequence similarity of STY3178 with 3DZA protein is nearly 40%. The homology model contains residues A^11^-Q^183^ excluding 21 N-terminal residues of the construct. At the C-terminal end of STY3178, there are 16 residues, for which no homology is obtained from the template. Thus, we add all these residues de-novo using Swiss-PdbViewer and minimize homology model for refinement. We perform MD simulation of this minimized homology model for 300 ns at 310 K. The MD simulated monomer is primarily helical containing total ten helices along with a two stranded antiparallel β-sheet as shown in Fig. 4a. The helices present in the structure are primarily forming two helix bundles where helices H2, H3, H7 and H8 form one bundle and helices H5, H6 and H10 forming the other.

### Molecular dynamic simulations at elevated temperatures

Our experimental data show that this protein unfolds at elevated temperature without change in its assembly. This leads to test the thermal stability of the monomer model from MD simulations. We simulate structures at 310 K, 350 K and 400 K. We find that the root means square fluctuation (RMSF) increases with temperature (Fig. 4b). In particular there are two regions which show enhanced RMSF with temperature. The first region is part of H3 and H4 along with the adjacent loop residues and the other region comprises of E1, H7 and the adjacent loops. The backbone dihedral (ϕ and ψ) distribution for majority of residues do not show change with increase in temperature (Figs 4c and S2). Only a handful of residues show sensitivity in the ϕ and ψ distribution, which includes N14, D17, N18 from H1; D69, W70 and N71 from H4; A132 from H6; S182, Q183, S184 and V185 from H9; V193, H195, A197 and A198 of H10. An example of such change is shown for residue W70 in Fig. 4d and S184 in Supplementary Fig. S3. The Ramachandran plots for these residues indicate structural changes from helix to loop and β-sheet (Supplementary Fig. S4).

The relative standard deviations at 350 K and 400 K with respect to those at 310 K, given by r_350_ = σ_350_/σ_310_ and r_400_ = σ_400_/σ_310_ respectively, are shown in Fig. 4e,f for different residues, colour coded according to their values. Residues showing r_350_, r_400_ ≤ 1 for both ϕ and ψ are shown in green in Fig. 4e,f. Majority of residues belong to the region 1 < r_350_, r_400_ < 4 with moderate fluctuations in either ϕ or ψ or both at 350 K and 400 K as shown in orange in Fig. 4e,f. The residues, having r_350_, r_400_ > 4 are marked in red which belong to the terminal helices H1, H9 and H10. Despite enhancement in fluctuations, the overall structure of STY3178 remains stable. To the contrary, we observe enhanced RMSF and loss of secondary structural element for lysozyme at 400 K from our simulation using the same force field as shown in Supplementary Fig. S5, in agreement with earlier report^35^.

## Discussion

**Figure 5:**
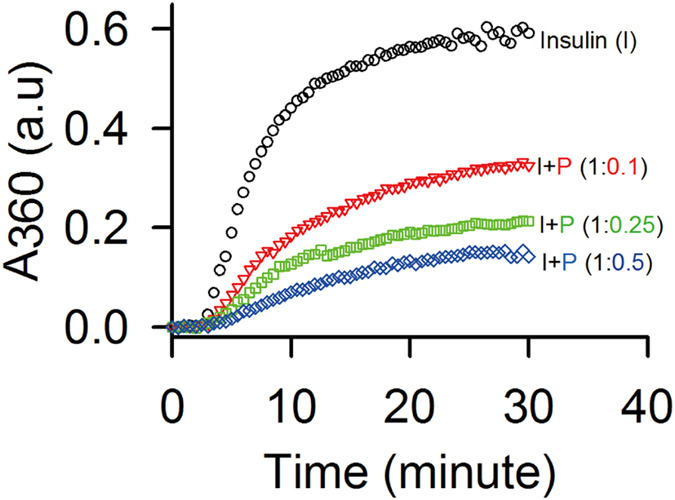
Chaperone activity assay. Absorbance at 360 nm (A_360_) for DTT induced insulin (I) aggregation (black) is represented. Insulin aggregation in presence of STY3178 (P) at molar ratio 1:0.1 (red), 1:0.25 (green) and 1:0.5 (blue) are shown.

We predict the function of STY3178 using bioinformatics tool MODexplorer^36^. The output results from MODexplorer server indicates nearly 50 proteins which show structural similarity >50% with STY3178. Among these 50 proteins most of the proteins have helices similar to STY3178. While some of the proteins with similar structures have no reported function, majority of them are either chaperones or assist chaperone activity. Our observations indicate that STY3178 may have chaperone activity. To verify such possibility, we monitor DTT induced aggregation of insulin B-chain in presence of STY3178. At 42 °C, we observe a steep rise in absorbance at 360 nm (A_360_) with time followed by a saturation platue (Fig. 5) as reported earlier^37^. In presence of STY3178 at same temperature, we find lower absorbance at 360 nm with time, indicating less aggregation of insulin B-chain in presence of DTT. This prevention of aggregation is dependent on the net amount of STY3178 present in the reaction mixture. Figure 5 shows the plot of absorbance at 360 nm (A_360_) versus reaction time (30 minutes) in absence (black) and presence of various STY3178 molar concentration ratios 0.1 (red), 0.25 (green) and 0.5 (blue) to insulin concentration. A systematic lowering of absorbance at 360 nm (A_360_) with increasing concentration of STY3178 demonstrates its capability to prevent insulin aggregation. Thus, *in vitro* we indeed observe that STY3178 is capable of showing chaperone activity.

STY3178 sequence from Gene bank (gene ID gi|16758993:c3049965-3049366) is used in signalP 4.1 server^38^ to identify any possible signal peptide sequence. The construct of STY3178 used in experiments do not contain 9 N-terminal residues. SignalP server predicts the first 12 residues in the N-terminal region of this protein as a signal peptide. Experimentally we observe that STY3178 is soluble in aqueous medium. This indicates that STY3178 cannot be a membrane embedded protein. Thus presence of predicted signal peptide in the N-terminus indicates a possibility of its localization in the periplasm. Exact cellular localization of this protein is not yet established. However, servers like Cello^39^,^40^ and LocTree3^41^ predicts its sub-cellular localization in the periplasm. There are many chaperones identified in the periplasm of bacteria like peptidyl-prolyl isomerases, disulphide bond isomerases etc^42^,^43^,^44^. Since ATP is absent in periplasm^42^, the chaperones from periplasm are capable of performing their activity without ATP assistance. The predicted sub cellular localization as well as ATP independent chaperone activity of STY3178 both suggests its localization in the periplasm.

## Conclusion

To summarize, our experimental studies reveal a reversible thermal unfolding and structural stability at elevated temperature for STY3178. The CD, steady state fluorescence and NMR data show that the protein is stable up to 45 °C and folds back even after heating at 80 °C. Further the time dependent CD measurements between 20 °C–70 °C show reversibility in unfolding with hysteresis. The kinetics of unfolding and refolding both follows the Arrhenius behavior with an activation energy barrier of ~246.9 kJ/mol and −58.66 kJ/mol, respectively. Our molecular dynamics simulation studies on the proposed model monomeric structure of the protein show stability at high temperature. Most importantly, we find an ATP independent chaperone activity capability of STY3178. This observation matches well with the bioinformatically predicted periplasmic localization of the protein. Our studies indicate that STY3178 may be an important protein through its chaperone activity for the bacterial cellular functions. Hence yfdX proteins need to be fully characterized to understand bacterial virulence.

## Methods

Plasmid pET28a carrying the desired gene *sty3178* is overexpressed in *E. coli* using 0.2 mM isopropyl-β-D-thiogalactoside for 4 hours in a shaker (Innova 42 New Brunswick Scientific) as reported earlier^2^. Protein extraction and purification are done following the earlier reported protocol^2^. All the reported measurements are carried out in a buffer containing 50 mM phosphate (pH 7), 250 mM NaCl and 1 mM PMSF. In all the experiments, the protein concentration is calculated in terms of monomer molecular weight (MW = 23107.7 Da).

### Circular Dichroism

All the circular dichroism (CD) measurements are carried out in Jasco J-815 CD spectrometer equipped with peltier temperature control unit (Jasco). The sample concentration and path length for all the experiments are 10 μM and 3 mm, respectively. Every CD spectrum is acquired in the far UV-region (200–250 nm). Unfolding of the protein is monitored in the temperature range of 20 °C to 70 °C upon increasing temperature with increments of 10 °C and equilibration of 10 minutes at each (30 °C, 40 °C, 50 °C, 60 °C and 70 °C) temperature. Similar to unfolding experiments, refolding of the protein is achieved by decreasing temperature from 70 °C with decrements of 10 °C till 20 °C (60 °C, 50 °C, 40 °C, 30 °C, and 20 °C). Background correction is done by subtracting the ellipticity value of buffer in the far UV-region for each experiment at that temperature.

The transition from folded state to unfolded state is monitored by measuring the ellipticity values at 222 nm [*θ*_222_] upon gradually heating from 20 °C to 70 °C. The fraction of the folded protein (*f*_*N*_) at each temperature is calculated using the expression^29^ *f*_*N*_ = (*θ*_*D*_ − *θ*_*T*_)/(*θ*_*D*_ − *θ*_*N*_), where *θ*_*N*_ and *θ*_*D*_ are the measured ellipticity of the folded and the unfolded protein respectively. *θ*_*T*_ is the ellipticity at each temperature. Heating rates 0.5 °C/min, 1 °C/min, 2 °C/min, 3 °C/min, 4 °C/min and 5 °C/min are used in CD experiments for probing the transition from folded to unfolded state. Similarly we use six different cooling rates (0.5 °C/min, 1 °C/min, 2 °C/min, 3 °C/min, 4 °C/min and 5 °C/min) for refolding the protein.

### Dynamic Light Scattering (DLS)

DLS data is acquired for the temperatures 20 °C, 30 °C, 40 °C, 50 °C and 60 °C using Nano-S Malvern instrument equipped with thermostat cell holder using methodology as described earlier^2^. Protein is equilibrated for 10 minutes at each temperature prior to acquiring data.

### Unfolding Kinetics

Thermal unfolding kinetics is followed from the change in ellipticity [*θ*_222_] upon heating the protein for 4 hours at temperatures 53 °C, 55 °C, 57 °C, 59 °C, 60 °C, 63 °C and 65 °C. The buffer is pre-equilibrated at each temperature prior to mixing with protein. The dead time of the whole process is less than 15 sec. *f*_*N*_ is calculated at different times and the time dependence has been fitted using single exponential decay equation, 
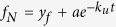
, where *k*_*u*_ is rate of unfolding and *t* is time. The activation energy (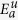) of unfolding has been estimated using Arrhenius equation, 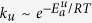, where R is the universal gas constant and T is the absolute temperature.

### Refolding Kinetics

The kinetics of refolding is calculated from the ellipticity change [*θ*_222_] of the unfolded protein at 222 nm. 10 μM protein is unfolded by heating at 70 °C for 10 minutes. The temperatures of the cell holder is set to six different temperatures (30 °C, 32 °C, 34 °C, 36 °C, 38 °C and 40 °C) allowing cooling of the unfolded protein and subsequent refolding. The time between the start of the experiment and rise of *f*_*N*_ is the lag time required for initiation of refolding. The lag time is primarily governed by the machine accuracy (99.5 sec-30 °C, 99.5 sec-32 °C, 99.5 sec-34 °C, 101 sec-36 °C, 99.5 sec-38 °C and 101 sec-40 °C). The entire set of data is collected for three repeated sets of experiments. We calculate the Δ*f*_*N*_ = *f*_*N*_ − *f*_*R*_, where *f*_*R*_ is the folded fraction from which the rise in *f*_*N*_ starts. The *f*_*R*_ values are typically small but non zero for different temperatures. Similarly, we define the rise time Δt by subtracting out the lag time. The plot of Δ*f*_*N*_ versus Δt show single exponential rise, fitted with 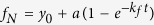 from which we estimate the rate of refolding (*k*_*f*_). The activation energy of refolding (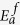) is obtained following Arrhenius equation by linear fitting of ln*k*_*f*_ with 1/T.

### Steady State Fluorescence

Fluorescence experiments are performed in Jobin Yvon Horiba Fluorolog attached with a peltier unit (Wavelength Electronics). Sample concentration of 10 μM and slit width of 2 nm is used for excitation at 280 nm. Sample is heated gradually at different temperatures starting from 20 °C to 70 °C with increments of 5 °C and equilibration of 10 minutes at each temperature (25 °C, 30 °C, 35 °C, 40 °C, 45 °C, 50 °C, 55 °C, 60 °C, 65 °C and 70 °C). Similarly, the same sample is allowed to cool back gradually from 70 °C to the starting temperature (20 °C) to monitor its refolding. The final emission spectra are obtained by subtracting the corresponding buffer spectrum (blank) from that of the sample.

### Nuclear Magnetic Resonance spectroscopy

The uniformly ^15^N-labeled protein sample is prepared by growing *E. coli* cells in M9 minimal media containing ^15^NH_4_Cl as the only source for nitrogen^2^. Extraction and purification protocols are same as done earlier^2^. NMR experiments are performed in a 600 MHz Varian spectrometer equipped with room temperature probe. ^1^H-^15^N heteronuclear single quantum coherence (HSQC) experiments are carried out for temperatures 25 °C, 30 °C, 35 °C, 40 °C and 45 °C.

Sample concentration used is 400 μM. The buffer condition used is 30 mM phosphate buffer (pH 7), 150 mM NaCl and 10% D_2_O. All HSQC experiments are performed with 64 scans and 128 number of points. Data processing and analysis are done using NMRPIPE^45^ and NMRVIEW^46^.

### Molecular model

Homology model of the tertiary structure of STY3178 is generated using the servers Phyre2^47^ and RaptorX^48^,^49^,^50^,^51^. Template used for the model generation is a yfdX protein from *K. pneumoniae* (PDB 3DZA) which is a homotetramer and contains four identical chains (A, B, C and D). Phyre2 used chain B and RaptorX used chain A of the template protein and generated identical models for STY3178. The homology model contains residues A^11^-Q^183^ only. There is no homology obtained for residues S^184^-H^199^. We generate a model for this region (S^184^-H^199^) using Swiss-PdbViewer^52^ by adding one residue at a time and minimizing thereafter. Hydrogen atoms are added to the final model containing residues A^11^-H^199^. The model is solvated using explicit solvent model in a rectangular parallelepiped water box with dimension 69.4 × 62.2 × 73 Å^3^ and minimized using NAMD^53^ for refinement after neutralizing with counter ions.

### Molecular dynamics simulation of protein model

NAMD^53^ is used for the molecular dynamics (MD) simulation of the minimized structure. CHARMM27^54^ force field and TIP3P^55^ water model is used for MD simulation performed at 1 atm pressure in isothermal-isobaric (NPT) ensemble following the standard protocol, with periodic boundary condition and 1 femtosecond (fs) time-step. Particle-mesh Ewald method is applied to deal with electrostatic interactions. Energy minimization of 10,000 steps is performed prior to 300 nanosecond (ns) simulation time with 29848 total number of atoms. The initial MD simulation is performed at 310 K temperature. An equilibrated structure from the 310 K ensemble is used for 350 K and 400 K temperature simulations. The root mean square deviation (RMSD) at all the temperatures is calculated with respect to the C_α_-atoms over all the trajectories up to 300 ns. We then estimate the root means square fluctuation (RMSF) of C_α_-atoms of each residue at temperatures 310 K, 350 K and 400 K over the equilibrated trajectories. The histogram distribution, Ramachandran plot and standard deviation (σ) of backbone dihedral angles ϕ and ψ of each residue is calculated over the trajectories 150–300 ns. The relative change in σ for each residue at 350 K (r_350_ = σ_350_/σ_310_) and 400 K (r_400_ = σ_400_/σ_310_) is then estimated with respect to that at 310 K.

MD simulation for the protein lysozyme (PDB 193L) is performed for 100 ns using the total number of 21002 atoms at 310 K and 400 K following the protocols mentioned above.

### Chaperone activity assay

Thermal aggregation of 50 μM insulin in presence of 20 mM DTT is monitored from the absorbance at 360 nm (A_360_). A 10 mm path length cell is used in BioSpectrometer (Eppendorf) for A_360_ measurement at 42 °C as described in earlier protocol^37^. The experiment is performed both in absence and in the presence of pure STY3178. Molar ratio of STY3178 used in the assay is 0.1:1, 0.25:1 and 0.5:1 with respect to insulin. The buffer used in the experiment is 50 mM phosphate (pH 7), 250 mM NaCl and 1 mM PMSF. Both the proteins are equilibrated at 42 °C for 10 minutes prior to addition of DTT.

## Additional Information

**How to cite this article**: Saha, P. *et al*. Reversible thermal unfolding of a yfdX protein with chaperone-like activity. *Sci. Rep.* **6**, 29541; doi: 10.1038/srep29541 (2016).

## Supplementary Material

Supplementary Information

## Figures and Tables

**Table 1:** Half denaturation (T_m_) and renaturation temperature (T_m_’) of protein STY3178 for different rates of heating and cooling.

Rate (°C/min)	T_m_ (°C)	T_m_’ (°C)
0.5	56.57	46.57
1	58.65	45.73
2	61.15	44.59
3	62.4	43.96
4	63.65	43.24
5	64.59	42.4
