# Supplementary material for: Reversible thermal unfolding of a yfdX protein with chaperone-like activity
Source: Sci Rep. 2016 Jul 11;6:29541. doi: 10.1038/srep29541 (PMC4941729; doi:10.1038/srep29541)

Supporting Information

Reversible thermal unfolding of a yfdX protein with chaperone-like activity

Paramita Saha, Camelia Manna, Jaydeb Chakrabarti and Mahua Ghosh*

Department of Chemical, Biological And Macromolecular Sciences, S. N. Bose National Centre for Basic Sciences, Block JD, Sector III, Salt lake, Kolkata 700098, India

Corresponding Author

Email: *mahuaghosh@bose.res.in, *mahua.ghosh@gmail.com

Figure S1. Plots of ln(k_u/T) and ln(k_f/T) as functions of 1/T.

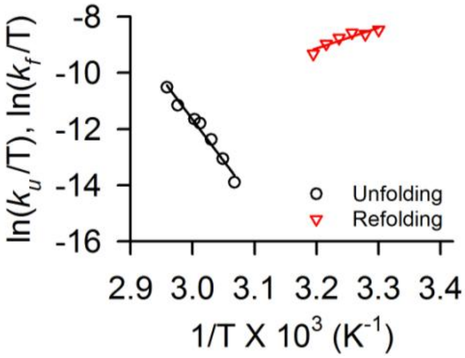

Figure S2. Backbone dihedral distribution of STY3178 residues at different temperatures from molecular dynamic simulation. The distribution of backbone dihedral angles ϕ and ψ for residues R41 and K112 at 310 K (circle), 350 K (inverted triangle) and 400 K (square) are shown in a to d.

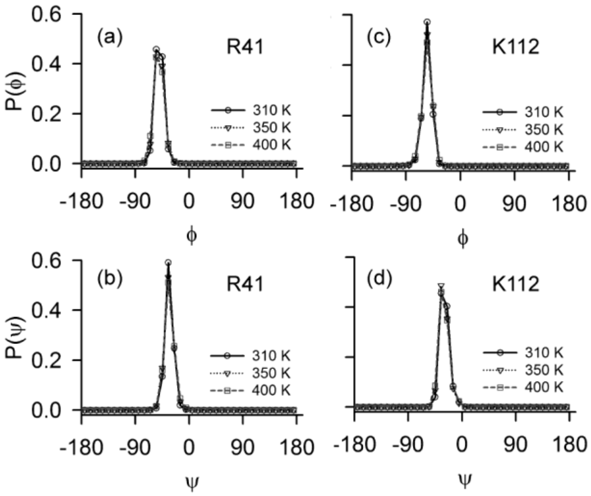

Figure S3. Dihedral angle distribution of residue S184 at elevated temperatures. (a) and (b) show the ϕ and ψ distribution, respectively, at 310 K (circle), 350 K (inverted triangle) and 400 K (square).

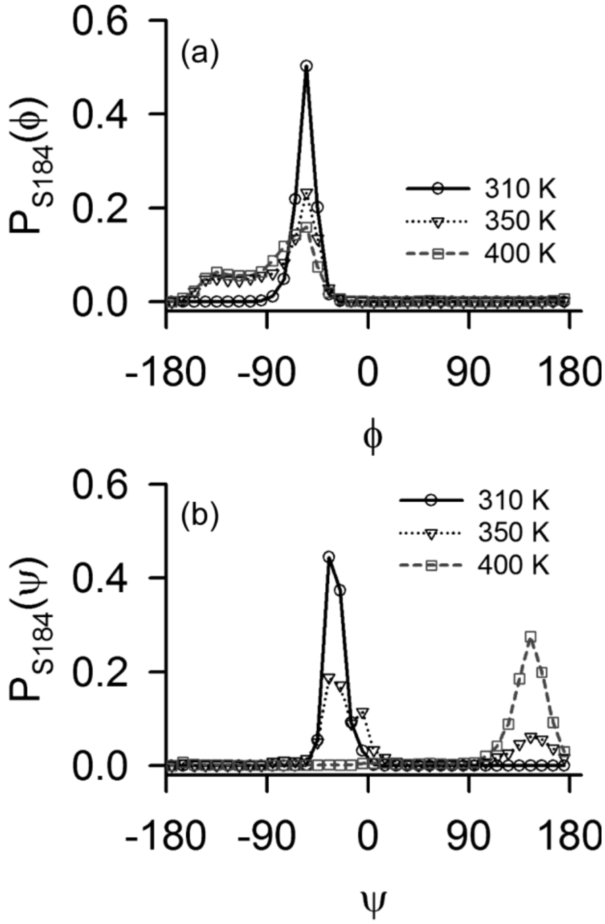

Figure S4. Ramachandran plot of affected residues of STY3178. The ϕ and ψ correlation plot for W70 and S184 are shown from (a) to (c) and (d) to (f), respectively for 310 K (circle), 350 K (inverted triangle) and 400 K (square).

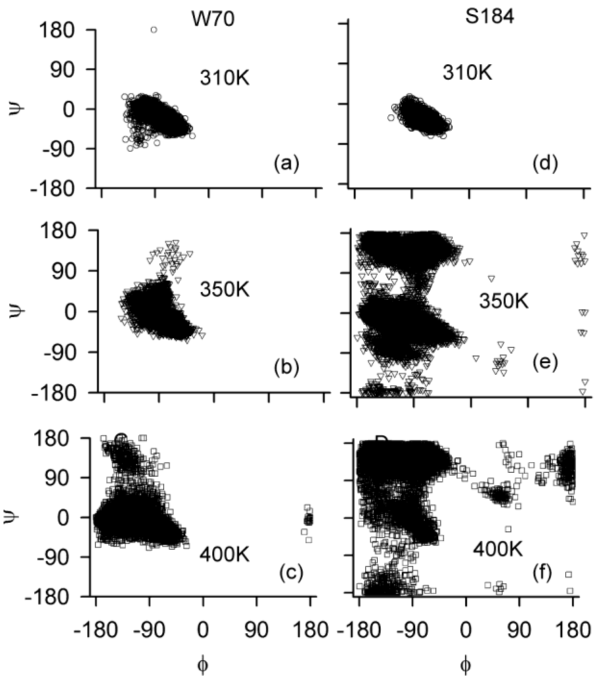

Figure S5. MD simulated Lysozyme structure at elevated temperature. (a) Shows the root mean square fluctuation of lysozyme residues over 50 ns to 100 ns for temperatures 310 K and 400 K. (b) The cartoon representation of 100 ns structure of lysozyme at 310 K (gray) and 400 K (black) is shown. The structural elements showing change at 400 K are circled in red.

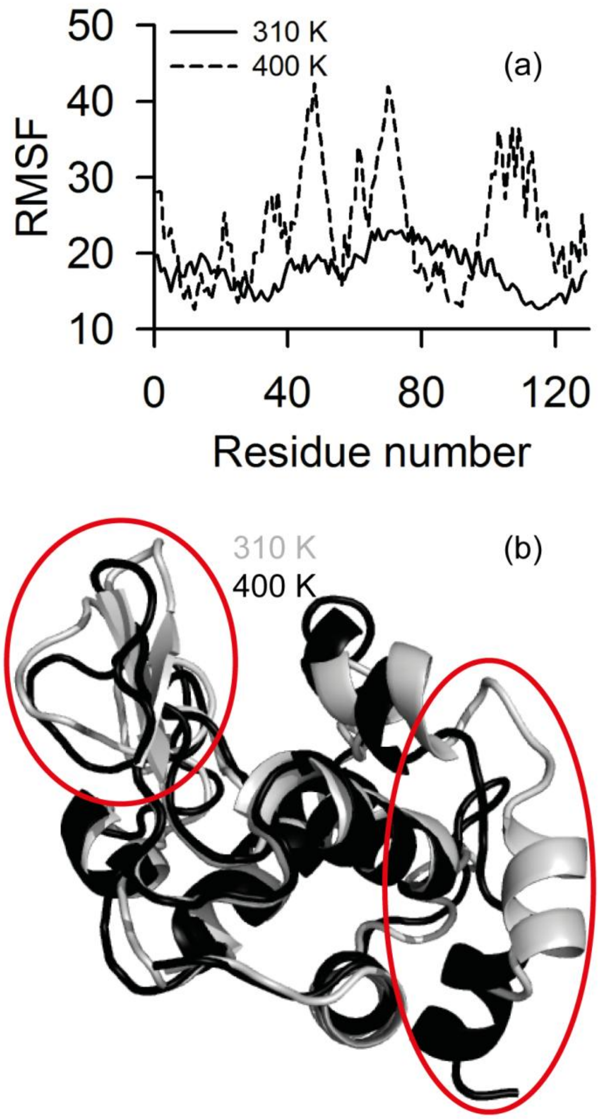

Supplement: Supplementary Information [file srep29541-s1.pdf]
